# Integrating oxytocin delivery through the vaccine cold chain system within the expanded programme of immunisation in Ethiopia: a case of evidence-informed decision-making

**DOI:** 10.1136/bmjgh-2025-022644

**Published:** 2026-05-29

**Authors:** Abdulhalik Workicho, Tsinuel Girma, Bereket Yakob, Zemzem Mohammed, Melkamu Ayalew, Mariamawit Asfaw, Mirkuzie Woldie

**Affiliations:** 1 Fenot Project, Harvard School of Public Health, Addis Ababa, Ethiopia; 2 Maternal Child and Adolescent Health Lead Executive Office, Ethiopia Ministry of Health, Addis Ababa, Ethiopia; 3 Discipline of Public Health Medicine, School of Medicine, University of KwaZulu-Natal, Durban, KwaZulu-Natal, South Africa; 4 Policy Strategy and Research Lead Executive Office, Ethiopia Ministry of Health, Addis Ababa, Ethiopia

**Keywords:** Decision Making, Health systems, Health policy, Maternal health, Immunisation

## Abstract

This paper shares Ethiopia’s experience and lessons in applying evidence-informed decision-making (EIDM) aiming to improve quality of oxytocin delivery at the point of care for managing postpartum haemorrhage. A 2022 survey of sampled health facilities in Ethiopia showed that a significant number of facilities did not store oxytocin in a refrigerator, and a little over half of them monitored the oxytocin temperature daily. In response, the Maternal and Child Health Department of the Ministry of Health engaged its Research Advisory Council (RAC) to examine the policy question. The RAC convened local researchers, programme managers and development partners to analyse the problem, synthesise global and local evidence and gather expert input using structured tools such as the GRADE (Grading of Recommendations Assessment, Development and Evaluation) Evidence-to-Decision framework. The process unfolded over 2 years, involving iterative engagement, validation workshops and leadership consultations. Ultimately, the Ministry endorsed integration of oxytocin into the vaccine cold chain, a decision shaped by both scientific evidence and contextual realities. This case highlights practical lessons on structuring and facilitating EIDM processes in health systems, including the value of institutionalised knowledge translation platforms, transparent evidence synthesis and sustained leadership engagement. The experience provides transferable insights for other low- and middle-income countries seeking to strengthen mechanisms that translate evidence into policy decisions.

**Summary box**

- The participatory approach by the Research Advisory Council (RAC) fostered trust among diverse stakeholders, mirrored the principles of transparency and created a platform for genuine co-creation of evidence.
- Through structured evidence synthesis and expert validation guided by the GRADE (Grading of Recommendations Assessment, Development and Evaluation) Evidence-to-Decision framework, the process ensured that recommendations were not only evidence-based but also practical and suited to the context.
- The leadership at the Ministry of Health, coupled with continuous dialogue among programme managers, experts and decision-makers, built political commitment and collective ownership, which were the key drivers for effective policy adoption.
- Despite the progress, sustaining financial and technical resources, managing competing programme priorities and accommodating the time-intensive nature of evidence-informed decision-making (EIDM) remain significant challenges that require deliberate attention.
- Moving forward, decentralising evidence-informed practices, strengthening operational capacity of the RAC, integrating EIDM into national performance systems and learning from best practices can solidify a culture of EIDM across all levels of the health system.

## Background

 Health policies are developed in response to emerging health system challenges. However, the quality of a policy heavily depends on the process by which it is developed.^1^,^6^ This is why structured policy development mechanisms, such as evidence-informed decision-making (EIDM), are necessary to reduce bias, increase transparency and promote stakeholder ownership.^7^ EIDM is globally recognised as an essential approach for improving health policy.^4 8^ The process is non-linear and should be systematic and transparent, applying structured and replicable methods to identify the best available evidence to be appraised and implemented.^9^,^11^ Additionally, it should integrate contextual factors, public opinion, professional experience and expertise, ensuring equity, feasibility, affordability, sustainability and acceptability of implementation.^12^,^15^

In many low- and middle-income countries (LMICs), the application of EIDM is inconsistent, often constrained by weak institutional mechanisms, fragmented communication and limited capacity.^1113 16^,^18^ Knowledge translation platforms (KTP) have emerged as a response to these gaps. Globally, networks such as EVIPNet^19^ create structured spaces where researchers, policymakers and civil society co-produce solutions, increasing transparency and accountability. In Ethiopia, the Maternal, Child and Adolescent Health Services Lead Executive Office (MCAH LEO) of the Ministry of Health (MoH) established the Research Advisory Council (RAC) in 2016. The RAC bridges the evidence and decision divide by bringing together the key actors, including researchers, programme implementers and policy and decision-makers. It mainly serves as an institutional mechanism for evidence synthesis, policy dialogues and recommendations. Members are volunteers drawn mainly from local universities and research institutions, partner organisations, including non-governmental organisations (NGOs) and United Nations agencies. The principles and approach of co-creation were implemented, with the utmost attention given to make the process transparent and mitigate biases. Structured tools such as the Grading of Recommendations Assessment, Development and Evaluation (GRADE) Evidence-to-Decision (EtD) framework^20^ guide the EIDM activities under the RAC. The teams also use their theoretical and practical experiences from academia and the programme.

Fenot project, a collaborative initiative between the Harvard T.H. Chan School of Public Health and the MoH, with financial support from the Gates Foundation, provides the logistical, technical and financial resources required for the successful implementation of the activities under the RAC.

## Rationale for this manuscript

While scholarly literature and global institutions advocate EIDM widely,^11^ the few published reports indicate that EIDM is context-dependent.^21^,^23^ Thus, documentation and reporting of policymakers’ and researchers’ practical experiences navigating the complex processes of ensuring EIDM contributes to learning between contexts. However, the evidence on implementation in specific contexts, particularly low-income settings, remains scarce.

This manuscript aims to document and share a practical case of using the RAC as a KTP to facilitate EIDM in Ethiopia. The evidence comes from our firsthand account of an EIDM case that showcases the context-specific process. Specifically, it captures how the RAC supported evidence synthesis and decision-making for integrating oxytocin delivery into the vaccine cold chain system.

### The problem: suboptimal oxytocin delivery at the point of care

The use of injectable oxytocin is the first-line in the prevention and treatment of postpartum haemorrhage (PPH), recommended by the WHO^24^ and is included in the list of essential medicines.^25^ The health system needs to ensure the quality of the oxytocin used at the point of care to effectively prevent PPH-related complications. However, studies in many LMICs revealed high rates of substandard oxytocin in healthcare settings. Poor-quality manufacturing and/or product degradation in the supply chain were the main reasons for poor quality oxytocin.^2426^,^28^ Additionally, many of the products in these settings may not be labelled or may not be stored at the appropriate temperature of 2–8°C, affecting their efficacy and potency. Quality oxytocin delivery in this manuscript is defined as ensuring that oxytocin reaching the point of care maintains its pharmacological potency through appropriate storage (2–8°C), reliable supply and timely availability during childbirth. Oxytocin is heat-sensitive and may lose effectiveness if stored above recommended temperature ranges. Ensuring a reliable cold chain for oxytocin therefore maintains its clinical effectiveness in preventing and managing PPH.^28^

A 2022 national maternal, newborn and child health commodity availability end-use verification assessment was conducted among 86 sampled health facilities and 16 Ethiopia Pharmaceutical Supply Service (EPSS) warehouses. The results showed that only 66% of health facilities stored oxytocin in a refrigerator, and only 53% of them monitored the oxytocin temperature daily. In contrast, all EPSS warehouses stored oxytocin in refrigerators and monitored temperatures daily. A national Services Availability and Readiness Assessment also reported that only 30% of the facilities had refrigerators. In these facilities, only 11% had maintained adequate temperature of refrigerators, that is, kept the temperature between 2–8°C.^29^

The Expanded Programme on Immunisation supports most refrigerators at health facilities in Ethiopia. But storing other medical products, like oxytocin, is not a common practice. Nevertheless, other countries maintain the stability of oxytocin by integrating into the vaccine cold chain.^30^ In most health systems, the adoption of innovations that are effective even in comparable settings is often complex and challenging. Coming across this challenge, the then Maternal and Child Health Directorate of the MoH requested its RAC to support with evidence the decisions to address the sub-optimal oxytocin delivery at the point of care.

The problem of suboptimal oxytocin delivery in Ethiopia represented a systemic failure beyond technical and physical storage issues. It has a significant link to Ethiopia’s broader struggle to reduce maternal mortality, with postpartum haemorrhage being the leading cause. The urgency was amplified by international benchmarks, with the WHO also recommending the integration of oxytocin delivery through national cold chain systems. Addressing the problem, therefore, had both national and global significance, demanding a decision that was evidence-driven, feasible and rapidly actionable.

### The evidence-informed decision-making process

The maternal and child health and immunisation thematic groups of the RAC were tasked to lead this work. The team applied a structured yet flexible approach while addressing this policy issue to frame the question and develop a guide to reviewing and synthesising the best available evidence. The guide was discussed to contain clear review/policy questions, the objective of the review, inclusion criteria and the steps to be undertaken in the evidence search, synthesis, communication and translation.

Stakeholder engagement was continuous, from technical working groups to high-level Ministry officials. Importantly, the process was iterative; early findings were discussed, critiqued and refined, fostering ownership and consensus. This iterative co-production of knowledge exemplifies how EIDM works in practice, where the interests of different programmes and groups intersect dynamically.

#### Problem analysis

The suboptimal oxytocin delivery problem was prioritised due to challenges of maintaining oxytocin quality at the point of care for effective prevention and management of PPH. The problem was first analysed and reframed into a clear policy question involving the staff of the MOH, maternal and immunisation services units and partners.

#### Evidence synthesis

Evidence synthesis was conducted through a rapid review of published and grey literature, complemented by expert consultations. An initial exploratory search was performed on PubMed to identify studies, reports and policy recommendations. Based on this preliminary review, three members of the team developed a focused strategy to guide further evidence identification. The larger team reviewed and finalised this strategy before applying it to search PubMed and Google Scholar for published and unpublished research, programme, practice and policy documents. Additionally, unpublished literature, programme and policy documents were gathered from online platforms like Google and organisational websites.

The literature search was conducted from 10 July to 28 July 2022. The search yielded 314 published and unpublished records which were imported to EndNote V.20 reference manager (Clarivate Analytics, Pennsylvania, USA), for organisation and removal of duplicates. Titles and abstracts were screened against the inclusion criteria to assess relevance to the policy question. Only 50 of the 314 met the inclusion criteria. The rest were excluded because they were duplicates or were not directly related to the main topic of interest. In a further full review, 20 of the 50 were excluded because they did not contain the relevant information. The study selection process is presented in the online supplemental material annex 1.

Therefore, the synthesis was conducted based on 30 individual documents. The reviewers charted the abstracted data into key areas, such as integrating oxytocin with the vaccine cold chain system, strengthening the existing standalone approach at the maternity unit of health facilities, introducing new heat-stable uterotonics, issues with feasibility and advantages and disadvantages of integration. The charted data were reviewed further by other members of the team and revised.

#### Recommendation formulation

After synthesising the evidence, the maternal and immunisation RAC subteams met on 16 November 2022 during a RAC review and planning session. Based on the review objective and policy question, the synthesis results were organised according to the two options for delivering quality oxytocin: through integration with the vaccine cold chain or strengthening the existing standalone system, whereby oxytocin is stored and delivered at maternity units. After a joint review of the review findings, the teams found the need for expert opinion for comprehensive evidence. This was done through an online survey of local maternal, child health, immunisation and cold chain experts. The GRADE EtD framework served as the main analytical tool, ensuring that criteria such as feasibility, equity, resource use and sustainability were considered during the acquisition of expert opinion. The survey tool contained respondents’ background characteristics and had 6 domains with 23 items on critical elements, including acceptability, feasibility, ease of implementation, ability to achieve the desired effects (effectiveness), likelihood of causing undesired effects (harms) and sustainability. The teams reviewed and revised the tool before testing it via the SurveyMonkey version on 9 February 2023. The survey participants were identified from 4 February to 13 February 2023, by the RAC team and collaboration with leaders at the MoH. Each item in the six domains was rated using a 5-point Likert scale, with response options ranging from 1 (strongly disagree) to 5 (strongly agree). For analysis, responses were dichotomised into ‘in favor of the option’ and ‘not in favor of the option’, where ‘strongly agree’ and ‘agree’ were classified as ‘in favor’, and all other responses as ‘not in favor’. Analysis was conducted separately for the integration and the standalone models. For the domain assessing undesirable effect, reverse coding was performed prior to analysis to ensure consistent interpretation of item directionality. The details of the online survey tool are provided in the Online supplemental material annex 2. The RAC provided equal opportunity to the immunisation and maternal health units and encouraged them to identify experts in their respective fields.

The initial SurveyMonkey ran from 14 February 2023 to 15 March 2023. It showed responses from 33 experts, with more respondents from maternal health than from child health and immunisation. Therefore, to reduce potential bias in the expert opinion acquisition, additional invitations were sent to child health and immunisation experts, as well as cold chain experts. Eventually, the number of respondents increased to 48, of which 40 provided complete information. The mean age of participants was 38 years, and they had an average of 13 years of professional experience. The survey was finally closed on 10 May 2023.

The RAC team met on 29 June 2023, to discuss the results of the survey. Responses were summarised by domain and presented graphically according to the proportion of responses favouring each option. A briefing document was then presented to the RAC and during the deliberations, the survey findings were considered alongside evidence from the literature review, global guidance and expert consultations. Using structured tools such as the GRADE EtD framework, the RAC integrated these multiple sources of evidence to assess policy options and their implementation implications. Though the results inclined towards the integrated oxytocin delivery model, many experts also voted in favour of a standalone option. Hence, the RAC decided to conduct a dialogue with experts from relevant stakeholders and reach a consensus on the desired option of the oxytocin delivery models. Accordingly, a half-day workshop was held among members of the maternal and child health and immunisation thematic groups to consolidate the findings and formulate the recommendations to be shared with the MCAH LEO.

#### Evidence-dissemination/communication

Communication of the evidence was made in two steps, targeting different audiences. The first one targeted maternal health and immunisation services desk team members, who primarily lead these programmes. In-person and virtual platforms were arranged to reach more participants. The RAC provided a detailed slide presentation of the entire process and the key recommendations. The main recommendation from the evidence was integration of the oxytocin delivery into the vaccine cold chain. Participants raised essential questions around the process, engagement of relevant stakeholders, feasibility of implementation, including any unintended consequences. Most of the concerns were already addressed by the evidence generated, and only a few were to be addressed by developing an implementation guide and standard operating procedures (SOPs). This step in the process has immensely helped in bringing the two programmes, maternal health and immunisation, to a similar understanding and a commitment to collaboratively support the integration process.

Integrating oxytocin delivery into the vaccine cold chain requires some changes in service delivery, a strong commitment and buy-in from all levels of the health system. Because of these reasons, the decision needed involvement and direction of MOH’s higher officials, in this case, of the state minister for the Programme. The team scheduled a meeting with the state minister and his senior advisors to present the whole process and evidence. The presentation included results of the iterations with key stakeholders, mainly the maternal and immunisation teams. It also highlighted solutions for potential challenges that may arise when implementing the integration. Interestingly, participants raised an interesting fact indicating that several health facilities have decided individually and integrated the storage of oxytocin into their vaccine cold chain. This was taken as additional local and practical evidence of the feasibility of oxytocin delivery integration. Finally, the state minister gave a clear and strong direction for integrating oxytocin delivery into the vaccine cold chain and developing an implementation guide and SOP to achieve this.

#### Evidence translation

**Figure 1:**
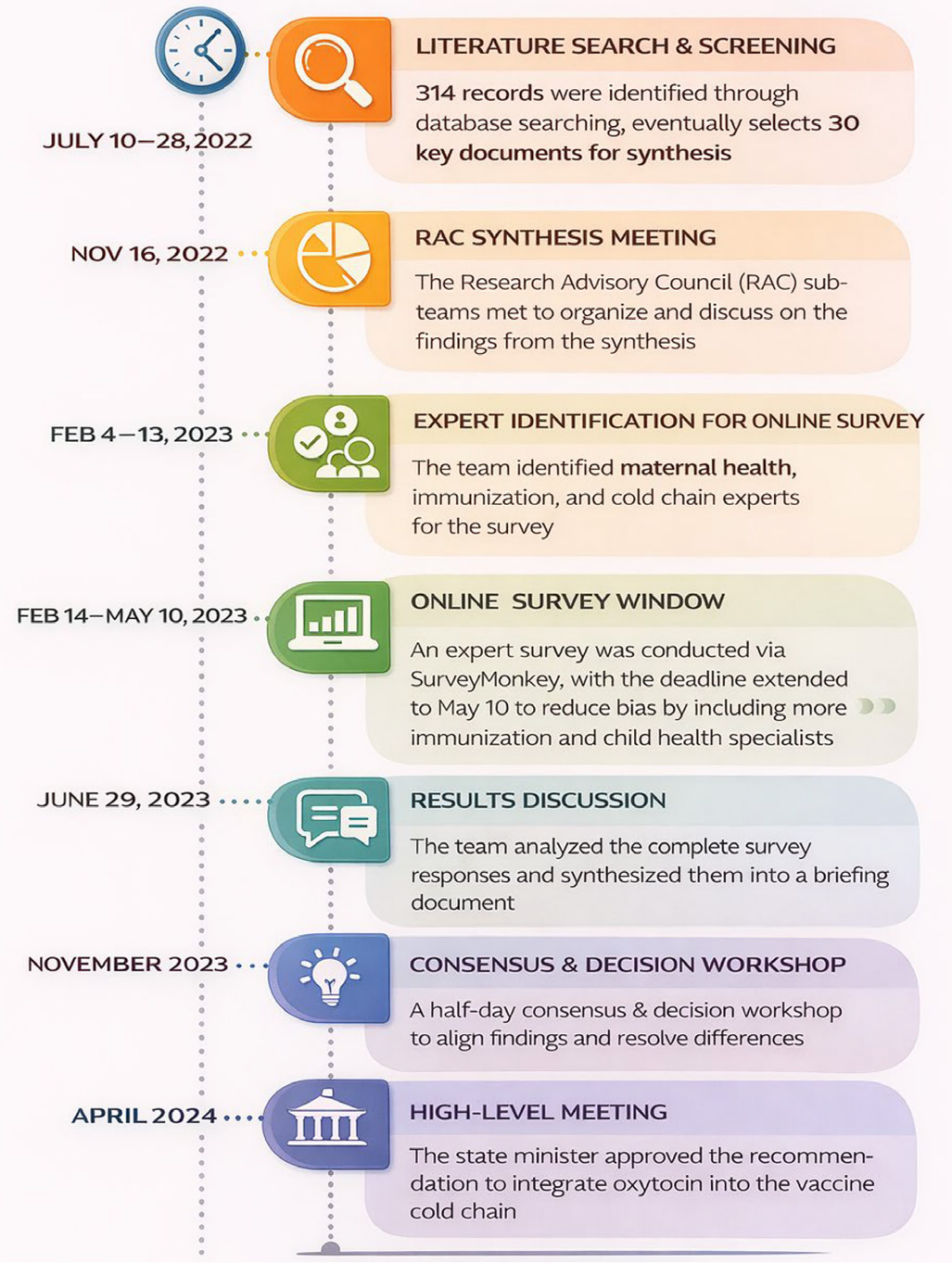
Key activity timelines in the evidence-informed decision-making process to integrate oxytocin in the vaccine cold chain system in Ethiopia.

Following this high-level decision, a team comprising maternal health and immunisation programme experts and researchers convened and prepared a draft outline of the implementation guide. Furthermore, they defined the content to be included in each section of the guide. Selected health facilities in four regions that were implementing their own form of integration were visited to document lessons, challenges and opportunities. These lessons informed the implementation guide on challenges that must be anticipated and addressed. Apart from documenting current integration practices in selected health facilities, the team also systematically collated expert opinions from maternal health, immunisation programmes and other relevant stakeholders to enrich the implementation guide. Once finalised, the implementation guide will be reviewed by the office of the state minister and approved. The approved version will serve as the reference document for designing capacity-building materials, training manuals, SOPs and monitoring and evaluation of the integration implementation. Figure 1 illustrates the main activities and timelines during this EIDM process.

## Lessons learnt

The case of oxytocin quality delivery integration in Ethiopia provided a lens through which to examine the application of EIDM frameworks in real-world health policy settings. Several important insights can be drawn from this case.

The participatory nature of RAC echoes the EVIPNet model’s emphasis on collaboration and transparency.^19^ This has also created an opportunity to co-create evidence and increase the contribution of key stakeholders. The co-creation brings synergy and mitigates potential conflicts during the implementation of the strategy. This EIDM process was primarily led by the RAC team, which gained trust and credibility in terms of being non-partisan to any of the programme units. This was critical since the immunisation team had some reservations out of genuine concerns when the integration was proposed. The RAC made a deliberate effort to be neutral and get opinions from a proportionate number of experts from both technical areas.

The structured review process was conducted through systematic evidence synthesis and validated with an expert opinion acquisition. This demonstrates a practical adaptation of the GRADE EtD framework.^10^ The standardised processes ensured that recommendations were not only evidence-based but also contextually feasible, acceptable and equitable. The systematic approach, documentation and frequent iterations have also enhanced the transparency of the process. The implementation planning that involved site visits, tool development and ongoing monitoring emphasises the importance of translation and sustainability. This aligns with EVIPNet’s guidance that evidence use should not end at policy endorsement but continue through implementation, monitoring and learning cycles.

The case illustrates how stakeholder dialogue can shape policy decisions. Dialogue sessions with programme managers, implementers, as well as higher officials, such as the state minister, created a feedback loop that allowed for clarification, consensus building and political buy-in, which are critical elements for policy adoption in a resource-constrained system. The strong leadership and ownership from the MOH enhanced credibility and confidence in the process. The leadership provided a fast and clear decision, which was made easier by well-synthesised evidence that was packaged and delivered suitable to the end user. The active involvement of high-level decision makers ensured political backing and signalled to all stakeholders that the process mattered. By involving immunisation and maternal health programmes as well as external experts, the process also reduced resistance and created a shared commitment.

Challenges included balancing competing programme priorities, which were mitigated through stakeholder engagement and transparent dialogue. On the proposal to integrate oxytocin into the vaccine cold chain system, the immunisation programme initially expressed concern that introducing oxytocin into the vaccine cold chain could compromise vaccine storage capacity, increase logistical complexity and create accountability issues across programmes. Conversely, maternal health experts emphasised that oxytocin requires cold-chain storage to maintain potency and that existing medicine supply chains often fail to guarantee optimal temperature control at the last mile. These concerns were addressed through structured evidence synthesis and stakeholder dialogue. Evidence on current cold-chain capacity, storage requirements and potential delivery models was reviewed, and expert opinions from both immunisation and maternal health programmes were incorporated. The analysis highlighted that oxytocin occupies minimal storage volume and could be accommodated within the existing cold chain with proper planning.

The process also explicitly considered mitigation strategies to address the identified risks. These included assessing cold-chain capacity at different levels of the health system, clarifying roles and responsibilities between maternal health and immunisation programmes and developing operational guidance to support coordinated implementation. In addition, the RAC recommended the development of a national implementation guideline to provide standardised procedures for storage, handling, accountability and monitoring of oxytocin within the existing cold-chain system. The recommendation also emphasised phased implementation and ongoing monitoring to ensure that vaccine storage integrity and immunisation programme performance would not be compromised. The communication package prepared for decision-makers therefore reflected both the potential benefits and the operational considerations associated with integration, along with proposed mitigation measures. This approach helped reconcile programmatic concerns and facilitated consensus among stakeholders.

Remaining challenges include ensuring sustainable financing for implementation of the recommendations and evaluating their effectiveness in bringing about the desired impact. An effective EIDM requires investment and time. Though most of the RAC members provided their professional service voluntarily, it has an opportunistic cost in terms of time and intellectual effort. A modest amount of finance is required to bring the teams together for workshops and consultative meetings. Moreover, the EIDM process takes time. In this particular case, the total process took over 2 years. It is mostly influenced by a combination of factors that include the nature of the policy issue, the need to engage a range of stakeholders, availability of evidence and the organisation’s decision-making culture or procedure. To enhance sustainability and routine application of EIDM in resource-constrained settings, several elements need to be considered. First, institutionalising knowledge translation platforms within the MoH or respective structures creates a formal mechanism for identifying policy questions, synthesising evidence and advising decision-makers as timely as possible. Second, using standardised tools and frameworks, approach, provide a structured process that can be applied across different policy issues easily. Third, sustained engagement of technical experts, programme managers and senior leadership builds trust and increases the likelihood that evidence will be translated into policy decisions within shorter time.

Globally, this case contributes to knowledge on how KTP can be institutionalised in low-resource settings, highlighting their importance in facilitating EIDM through mapping stakeholders, fostering trust and engaging leaders proactively. It also contributes to the growing body of evidence that KTP are not optional add-ons but essential health system functions.

## Conclusion

The availability and intentional use of institutionalised KTPs, like the RAC, can foster a culture of evidence use and identify and prioritise policy questions. Going forward, such practices of EIDM need to be decentralised to subnational health bureaus. To further advance the institutionalisation of EIDM in Ethiopia, a multifaceted strategy is essential. Strengthening the operational capacity of the RAC and similar other platforms through sustained financial and technical support will help ensure continuity, responsiveness and the ability to address emerging policy needs. Decentralising EIDM by expanding training to all regional health bureaus is also critical for embedding evidence use at every level of the health system, thereby promoting consistency in decision-making and implementation. Moreover, integrating EIDM indicators into national health system performance frameworks can help systematic tracking of evidence adoption, utilisation and impact. Developing national knowledge translation toolkits rooted in globally recognised frameworks such as GRADE EtD can provide a standardised guide for future policy cycles, enhancing both the quality and efficiency of evidence translation. Finally, fostering peer learning and collaboration by sharing Ethiopia’s EIDM experience with similar countries can generate reciprocal insights and accelerate regional progress toward data-driven health governance.

## Supplementary material

10.1136/bmjgh-2025-022644 online supplemental file 1

## Data Availability

All data relevant to the study are included in the article or uploaded as supplementary information.
