# Supplementary material for: Integrating oxytocin delivery through the vaccine cold chain system within the expanded programme of immunisation in Ethiopia: a case of evidence-informed decision-making
Source: BMJ Glob Health. 2026 May 29;11(5):e022644. doi: 10.1136/bmjgh-2025-022644 (PMC13223645; doi:10.1136/bmjgh-2025-022644)
Supplement: online supplemental file 1 [file bmjgh-11-5-s001.docx]

Supplemental material: PRISMA diagram and online survey tool used for acquisition of expert opinion as part of the evidence synthesis.

Annex I: PRISMA Flow Diagram

**Identification of studies via other methods**

**Identification of studies via databases and registers**

Records identified from organizational websites and program reports: (n = 20)

Records removed *before screening*:

Duplicate records and documents not related to the topic removed (n = 264)

Records identified from*:

Databases (n = 294)

**Identification**

Reports sought for retrieval (n = 12)

Records screened (n =38 )

Records excluded (n = 20)

Reports assessed for eligibility (n = 12)

Reports sought for retrieval (n =18)

Reports not retrieved (n = 0)

**Screening**

Reports assessed for eligibility (n = 18)

Reports excluded: (n=0)

Studies included in review

(n =18 )

Reports of included studies (n = 12)

Total=30

**Included**

Annex II: Tool used for acquisition of expert opinion as part of the evidence review and synthesis

Postpartum hemorrhage (PPH) remains the leading cause of maternal mortality in Ethiopia. A recent systematic review reported that hemorrhage had become a more prominent cause of maternal death, contributing 29.9% (95%CI: 20.28%-39.56%) of the direct causes. The World Health Organization (WHO) has recommended using oxytocin to prevent and manage PPH. This recommendation has been accepted and implemented by many countries, including Ethiopia. However, oxytocin is temperature sensitive and requires storage between 2 and 8^0^C. Due to the lack of an appropriate cold chain to maintain the integrity of this drug in the country’s health system, there are concerns about the effectiveness of oxytocin used for the prevention and treatment of PPH. The MNCH End Use Verification (EUV) assessment in Ethiopia, done by GHSC-PSM, has shown that significant proportion of facilities do not monitor the temperature of oxytocin in cold chain storage; and near to 16% of facilities do not store oxytocin in a refrigerator. Most refrigerators available at health facilities are supported by the Expanded Program on Immunization (EPI). Hence, other products, like oxytocin, might not be allowed to be stored in these refrigerators.

On the other hand, Ethiopia has a reasonably functional cold chain system for the Extended Program on Immunization (EPI). EPI cold chains in other countries have been used to maintain the stability of oxytocin by integrating the drug in the cold chain. However, health systems are too complex to enable the simple adoption of an innovation used elsewhere in the world. In view of the existing critical problem in maintaining the potency of oxytocin, the Maternal and Child Health Directorate of the Ministry of Health has requested the Maternal and PMTCT and Child Health and Immunization thematic groups of its Research Advisory Council (RAC) to conduct an in-depth analysis of options that will help to maintain the quality of oxytocin all along with the health system. The MoH wants evidence to know the relative advantages and disadvantages of the options, including human resource, feasibility, safety, stakeholders’ buy-in, and cost-effectiveness considering contextual factors. The Directorate plans to use the evidence as input to guide policy decisions that help maintain the quality of oxytocin used to manage PPH in health facilities.

Hence, this brief expert opinion-seeking tool is prepared to obtain more practical inputs from experts and stakeholders involved in the national programs that involve management of the cold-chain system including the maternal and child health units and departments. Based on the systematic review findings conducted by the two thematic groups of the RAC, two options i.e., either integrating oxytocin with the EPI cold chain or “strengthening/establishing the oxytocin storage at maternal units. were proposed to solve the existing problem of oxytocin management and ultimately improve maternal health outcomes. Dear expert, participation in this survey is voluntary. Your participation will have a huge contribution, and your best expert opinion is badly needed to develop an impactful national policy. Completing this tool may take only 10-15 minutes. The tool will be administered based on a 5-point Likert scale **[1: Strongly**

**disagree, 2: Disagree, 3: Neutral, 4: Agree, and 5: Strongly Agree]** to get the expert judgment on the feasibility, cost-effectiveness, acceptability, and sustainability among others of the two options.

| **Criteria** | **Possible Options** | | **Remark** |
| --- | --- | --- | --- |
|  | ***Integration with the existing EPI cold chain system: e.g. transportation,***  ***storage, and management*** | ***Strengthening/establishing Oxytocin storage at the maternity***[***^2^***](#bookmark0) ***unit*** |  |
| **Desirable effects** | 1 2 3 4 5 | 1 2 3 4 5 |  |
| Potential implementation in the long-term | 1 2 3 4 5 | 1 2 3 4 5 |  |
| The implementation of this option improves the quality of  oxytocin. | 1 2 3 4 5 | 1 2 3 4 5 |  |
| The implementation of this option improves cost-  efficiency. | 1 2 3 4 5 | 1 2 3 4 5 |  |
| The implementation of this option improves the efficiency  and effectiveness of the supply chain | 1 2 3 4 5 | 1 2 3 4 5 |  |
| This option can be an immediate solution to solve the  current problem of oxytocin (losing its potency). | 1 2 3 4 5 | 1 2 3 4 5 |  |
| **Undesirable effects** | 1 2 3 4 5 | 1 2 3 4 5 |  |
| Implementing this option will compromise the quality and  efficacy of vaccine products. | 1 2 3 4 5 | 1 2 3 4 5 |  |

2 *This refers to storage of oxytocin and also assuming other birth dose antigens in a standard cold chain equipment at maternity ward/delivery site. This starts with revitalization of HFs with equipment/fridges and continue the effort to establish new ones in others. In this respect, there is a shared responsibility and integrated functions such as capacity building, vaccine management and forecasting, preventive maintenance and repair of cold chain equipment with the EPI team in that facility. I think it is good to clarify/elaborate this option for better judgement and scoring of the listed criteria.*

| Implementation of this option will introduce programmatic  (medical) errors. | 1 2 3 4 5 | 1 2 3 4 5 |  |
| --- | --- | --- | --- |
| Implementation of this option will bring coordination  challenges. | 1 2 3 4 5 | 1 2 3 4 5 |  |
| **Demands intensive resources** | 1 2 3 4 5 | 1 2 3 4 5 |  |
| The implementation of this option demands more human  resource | 1 2 3 4 5 | 1 2 3 4 5 |  |
| The implementation of this option needs new technology | 1 2 3 4 5 | 1 2 3 4 5 |  |
| The implementation of this option needs new/additional  equipment | 1 2 3 4 5 | 1 2 3 4 5 |  |
| The implementation of this option demands a  new/additional transport system. | 1 2 3 4 5 | 1 2 3 4 5 |  |
| The implementation of this option demands new/additional  storage space. | 1 2 3 4 5 | 1 2 3 4 5 |  |
| The implementation of this option incurs additional  /operational cost. | 1 2 3 4 5 | 1 2 3 4 5 |  |
| **Feasibility to implement** | 1 2 3 4 5 | 1 2 3 4 5 |  |
| The implementation of this option would be feasible in  terms of health care workers (may/may not need additional human resources). | 1 2 3 4 5 | 1 2 3 4 5 |  |
| The implementation of this option would be feasible in terms of logistic management (in view of the existing  system). | 1 2 3 4 5 | 1 2 3 4 5 |  |
| **Acceptability of the option** | 1 2 3 4 5 | 1 2 3 4 5 |  |
| The implementation of the option is acceptable by health  care providers | 1 2 3 4 5 | 1 2 3 4 5 |  |

| The implementation of the option is acceptable by health  system managers | 1 2 3 4 5 | 1 2 3 4 5 |  |
| --- | --- | --- | --- |
| The implementation of the option is acceptable by logistic  managers | 1 2 3 4 5 | 1 2 3 4 5 |  |
| The implementation of the option is acceptable by higher-  level decision-maker | 1 2 3 4 5 | 1 2 3 4 5 |  |
| **Sustainability** | 1 2 3 4 5 | 1 2 3 4 5 |  |
| It can be easily adapted into the existing system. | 1 2 3 4 5 | 1 2 3 4 5 |  |
| The existing policy environment is supportive of this option. | 1 2 3 4 5 | 1 2 3 4 5 |  |
